# Neuropsychiatric Manifestations, Reduced Self-Esteem and Poor Quality of Life in Children and Adolescents with Neurofibromatosis Type 1 (NF1): The Impact of Symptom Visibility and Bullying Behavior

**DOI:** 10.3390/children10020330

**Published:** 2023-02-09

**Authors:** Nicola Davide Cavallo, Gianpaolo Maggi, Francesco Ferraiuolo, Anna Sorrentino, Silverio Perrotta, Marco Carotenuto, Gabriella Santangelo, Claudia Santoro

**Affiliations:** 1 Department of Psychology, University of Campania “Luigi Vanvitelli”, 81100 Caserta, Italy; 2 Department of Women’s and Children’s Health, and General and Specialized Surgery, University of Campania “Luigi Vanvitelli”, 80138 Naples, Italy; 3 Department of Mental and Physical Health and Preventive Medicine, Child and Adolescent Neuropsychiatry Clinic, University of Campania “Luigi Vanvitelli”, 80131 Naples, Italy

**Keywords:** neurofibromatosis type 1 (NF1), children, adolescents, bullying, victimization, psychological symptoms

## Abstract

Neurofibromatosis type 1 (NF1) is an autosomal dominant condition, associated with neurocutaneous manifestations and neuropsychiatric manifestations. The present study explored the prevalence of bullying/cyberbullying behaviors and victimization behaviors in a cohort of children and adolescents with NF1. Possible gender differences and predictors of psychological symptoms, quality of life (QoL), and self-esteem were also examined. Thirty-eight school-aged participants with NF1 completed a psychological evaluation designed to assess anxiety and depression symptomatology, QoL, self-esteem, and the prevalence and extent of bullying/cyberbullying and victimization behaviors. We found that our participants frequently reported victimization behaviors rather than bullying/cyberbullying ones. Moreover, participants complained of depressive and anxiety symptomatology together with reduced self-esteem, and low psychosocial quality of life, with females reporting more severe performances than males. Furthermore, we found that reduced self-esteem was associated with more visibility of the NF1 symptoms, and victimization behaviors were found to mediate the relationship between anxiety and psychosocial QoL. Our findings indicated the presence of a maladaptive loop in children and adolescents with NF1 patients characterized by psychological symptoms, unfavorable self-perception, low self-esteem, and psychosocial difficulties that might be worsened by experiencing victimization behaviors. These results suggest the need to use a multidisciplinary approach in the diagnosis and treatment of NF1.

## 1. Introduction

Neurofibromatosis type 1 (NF1) is an autosomal dominant condition, with a prevalence of 1 in 3000 live births. It is due to heterozygous pathogenetic variants in the homonym gene codifying for the ubiquitous protein neurofibromin which is a negative regulator of the RAS/MAPkinase pathway [1]. NF1 can be sporadic or familial depending on whether the NF1 variant is de novo or inherited from an affected parent. NF1 is a multisystemic disorder since it may involve nervous, skeletal, cardiovascular, and endocrine systems, and may present an unpredictable phenotype, age-dependent appearance of key features, and very few genotype–phenotype correlations found to date. Revised diagnostic criteria for NF1 have been provided by Legius and colleagues [1] to help clinicians in identifying and differentiating NF1 and Legius syndrome that show phenotypic overlap in young patients with pigmentary findings.

Children and adolescents with NF1 may develop tumors primarily involving the nervous system including plexiform, cutaneous, and nodular neurofibromas, which have an aesthetic and potentially disfiguring effect. From the aesthetic point of view, patients tend to present facial dysmorphic features similar to those of the Noonan syndrome, scoliosis, thoracic abnormalities, a lower height than expected, macrocephaly, and also segmental overgrowth (elephantiasis neuromatosa) [2,3].

Along with these clinical manifestations, impairment in language and visuospatial abilities, executive dysfunctions, attention difficulties as well as poor emotional and social skills have been reported in NF1 [4]. Moreover, other neurodevelopmental disorders such as learning disabilities, often in the realm of reading, and Attention-Deficit/Hyperactivity Disorder (ADHD) may appear in comorbidity [5,6]. All these conditions are not mutually exclusive and can co-occur leading to challenging neurocognitive profiles of children with NF1 for diagnostic and therapeutic purposes. 

Indeed, adult NF1 patients with neurofibromatosis reported significantly more behavioral symptoms, higher levels of perceived stress, and lower levels of self-esteem as compared with the general population [7]; depression and anxiety symptoms are very frequent [7,8], and more severe than in patients with life-threatening diseases such as cancer [9,10]. In addition, NF1 is linked to poor quality of life (QoL) subdomains such as physical function, bodily pain, mental health, social function, and general health in adults and young patients [11,12,13] and women reported worse NF1-related QoL than men [14]. Psychological symptoms such as anxiety, depression, inattention, impulsivity, internalizing and externalizing disturbances, as well as difficulties in socializing, have also been reported in children and adolescents with NF1 [15,16,17].

Despite some strong evidence suggesting that children affected by NF1 may have low-level QoL compared to the general population [11,16,18,19], a recent review by Sanagoo and colleagues [12] failed to confirm this result. Thus, further studies should clarify the impact of NF1 on patients’ QoL.

Considering that physical manifestations negatively impact on psychosocial and emotional adaptation [20], NF1-related features and complications (i.e., cutaneous neurofibromas and severe scoliosis) may negatively affect the psychological wellness of individuals with NF1 both in adulthood and childhood. 

Cutaneous neurofibromas, particularly those in visible areas of the body such as the face and upper limbs, seem to be associated with reduced QoL [21]. However, a study by Cipolletta and colleagues [11] examining psychosocial functioning, QoL, and the self-image of children with NF1 indicated that poor QoL and distorted self-image could not be explained by the mere presence of aesthetic malformations without considering the possible confounding effects of anxiety and depression. Therefore, clinicians should evaluate the occurrence of psychological symptoms due to their impact on QoL [22].

Experiencing bully–victim behaviors might also increase the severity of psychological symptoms [23,24]. Bullying is a complex psychosocial phenomenon defined as repeated exposure to verbal, physical, and/or socially harmful behaviors by other individuals [25] that due to the development of new communication technologies and their use among youngsters can also occur in cyberspace. In addition to its prolonged duration over time, intentionality and the imbalance of power between the bully and the victim are two key characteristics of bullying [26]. In fact, the phenomenon of bullying can be analyzed from two different perspectives: one of the bullies and that of the victim. Bullies usually are physically stronger than their victims and are socially valued by their followers, while, in contrast, the victims are weaker, insecure, with low self-esteem, and tend to avoid conflict [27]. Then, victimization behaviors represent the other side of the coin and are associated with the development of internalizing symptoms such as anxiety and depression [28].

Furthermore, the emergence of new technological advancements and communication channels led to the advent of new forms of bullying. The term “cyberbullying” refers to aggressive actions carried out through technology-mediated communication (i.e., internet or mobile phones) that are intentional and practiced by a group or individual against one or more victims [29]. Their characteristics are such that these behaviors can be carried out at any time and from any place (even if the victim is not online) and that the perpetrators may reach a much wider audience whilst remaining anonymous [30,31]. In addition, cyberbullies do not necessarily have certain personal or physical traits and qualities such as physical dominance or the social competence required for traditional bullying and may perpetrate their bullying behaviors simply through the outward expression of hate using social media. Nevertheless, cybervictimization’s consequences on the victim’s health are as severe as face-to-face bullying leading to depressive and somatic symptoms, higher levels of stress, and suicidal ideation [32,33,34].

However, few studies have investigated the occurrence and severity of bullying behaviors in the NF1 population. A study by Holland and colleagues [35] using an NF1 school-aged cohort found that about 62.0% of participants have been bullied at least once in the past year and 13.6% reported being victimized every week, whereas a study by Hummelvoll and Antonsen [36] in young adults with NF1 provided evidence of frequent victimization behaviors over the course of the life. Moreover, a study by Stavinoha and colleagues [37] exploring the possible risk factors for bullying in NF1 revealed that the occurrence of ADHD and/or information processing difficulties contributes to social difficulties representing a risk factor for social victimization in children with NF1. Nevertheless, to the best of our knowledge, no study has explored frequency, severity, and psychological consequences in school-aged individuals with NF1.

To date, the present study aimed at exploring the prevalence and the severity of bullying/victimization and cyberbullying/cybervictimization behaviors by analyzing possible sex differences and risk factors in a cohort of children and adolescent outpatients with NF1. Moreover, we aimed at exploring possible consequences of bullying behaviors (in all its manifestations) in terms of psychological symptoms (i.e., depression and anxiety), QoL, and self-esteem. 

## 2. Materials and Methods

### 2.1. Subjects and Procedure

Thirty-eight consecutive participants were recruited at the Pediatric Neurofibromatosis Referral Center of the University of Campania “Luigi Vanvitelli”. All participants met the following criteria: (i.) a diagnosis of NF1 established according to the revised criteria published by Legius and colleagues [1]; (ii.) absence of intellectual disability according to the Diagnostic and Statistical Manual of Mental Disorders 5 [38]; (iii.) absence of other neurological or disabling conditions. Clinical data about familiarity with NF1 disease, the number of hospitalizations (both in and out-patient care), the number of surgical and pharmacological therapies, and neuropsychiatric comorbidity were collected for each patient through the clinical note. 

### 2.2. Pediatric and Psychological Assessment

At first, participants underwent a pediatric visit that lasted approximately 30 min and subsequently participated in the psychological assessment in a single session that lasted approximately 90 min.

Disease severity and the extent of symptom visibility were evaluated according to a modified version of Ablon’s scoring system [39] (see Table S1 (Supplementary Materials)). Specifically, two blinded clinicians with experience in pediatric care of NF1 (CS, SP) evaluated Ablon’s clinical score separately and discussed divergent results. 

All participants underwent: (i) the Children’s Depression Inventory−2 [40] to identify depressive symptomatology; (ii) the Revised Children’s Manifest Anxiety Scale −2 [41], a self-report tool to assess anxiety symptoms; (iii) the Rosenberg Self-Esteem Scale [42] to assess the attitude and consideration that a person has of himself; (iv) the psychosocial sub-scale of the Pediatric Quality Of Life Inventory self-report [43] in order to measure the psychosocial QoL in clinical and non-clinical children and adolescents; (v) a modified version of Olweus Bully/Victim Questionnaire [44] to specifically evaluate the presence and the severity of bullying and victimization behaviors; (vi) the Students’ Needs Assessment Survey [45] to detect and characterize the type of cyberbullying and cybervictimization behaviors.

Custodial adults and children’s written informed consent were obtained to allow the data collection and its use for research purposes. Participants were informed that participation in the psychological assessment was on a voluntary basis and that if they wanted, they could withdraw from the face-to-face interview at any time. None of them withdrew from the study. Participants were also reassured about the protection of their privacy. Data were collected and stored under law 196 of 30 June 2003, art. 13, and subsequent amendments; information provided by participants was used only for scientific and statistical purposes.

The study was carried out in 2019, conducted following the ethical standards of the Declaration of Helsinki and its later amendments, and approved by the Ethics Committee of the University of Campania “Luigi Vanvitelli”.

### 2.3. Statistical Analysis 

Demographic, clinical, and behavioral characteristics of the sample were compared between male and female participants using the non-parametric Mann–Whitney *U* test. 

We evaluated the association of anxiety, depression, self-esteem, and psychosocial QoL with demographic, clinical, and behavioral variables in the whole sample carrying out several multiple regression analyses: demographic (i.e., sex and age), clinical (i.e., symptoms’ severity and visibility), and behavioral (i.e., psychological symptoms and bully/victimization behaviors) variables were entered as independent variables and anxiety, depression, psychosocial QoL, and self-esteem scores as dependent ones. 

Furthermore, in order to explore whether and how victimization behaviors mediated the relationship between psychological symptoms (i.e., depression and anxiety) and participants’ psychosocial QoL, we carried out a parallel mediation analysis entering the score on psychosocial QoL as the dependent variable, depression and anxiety scores as predictors, and scores on tests evaluating the level of victimization/cybervictimization behaviors as parallel mediators. This analysis was performed using SPSS Macro PROCESS [46] and bootstrapping procedure with 5000 samples and replacement from the full sample was applied to construct bias-corrected 95% confidence intervals (hereafter 95% CI; LL = lower level of the confidence interval; UL = upper level of confidence interval).

The critical alpha level for all analyses was set at 0.05. All analyses were performed with IBM SPSS-20.

## 3. Results

In this study, we enrolled 38 NF1 outpatients (17 females and 21 males) aged between 7 and 16. The sociodemographic and behavioral characteristics are shown in Table 1.

We found 20 participants (52.6%) reported victimization behaviors and 11 participants (28.9%) reported bullying behaviors, once or twice in the past six months. Using the more restrictive criteria by Solberg and Olweus [47], we observed victimization behaviors in nine participants (23.7%) and bullying behaviors in three (7.9%). Regarding cyberbullying/cybervictimization we found 12 (31.6%) participants had experienced cybervictimization behavior and 6 (15.8%) cyberbullying behavior. 

In addition, anxiety symptoms, assessed by the RCMAS-2 total score, ranged from moderate to severe in seven participants (18.4%); depressive symptomatology, evaluated by CDI-2 total score, was clinically significant in two participants (5.3%); self-esteem, evaluated by RSES, was significantly low in five participants (12.8%) within our sample. 

Furthermore, we found the mean score of psychosocial QoL, assessed by PedsQoL, in our sample was 74.2 (SD = 14.86).

Regarding disease severity and symptom visibility, evaluated by Ablon’s modified scale, we found that 14 participants (36.8%) reported mild severity, 19 participants (50%) reported moderate severity and 5 (13.2%) reported severe symptomatology of NF1 disease. Instead, symptoms’ visibility was mild in 6 (15.8%) participants, moderate in 22 (57.9%), and severe in 3 (7.9%) of them. We found that seven (18.4%) participants did not report any NF1 visible manifestation.

The comparison between females and males showed that female participants reported more severe anxiety and depression symptoms, lower self-esteem, lower psychosocial QoL, more frequency of victimization behaviors, more use of pharmacological therapies, and more frequency of day hospital visits, compared to male participants (see Table 1).

### 3.1. Sociodemographic and Behavioral Predictors of Psychological Variables and Psychosocial QoL

As for anxiety symptoms, linear regression analysis indicated that higher anxiety was associated with the female sex (*B* = 4.275, *t* = 2.066, *p* = 0.048), more cybervictimization behaviors (*B* = 1.310, *t*= 2.135, *p* = 0.041), more severe depressive symptomatology (*B* = 0.429, *t* = 2.548, *p =* 0.016), and lower self-esteem (*B* = −0.686, *t* = −2.904, *p* = 0.007). At the same time, more severe depression was related to more severe anxiety (*B* = 0.427, *t* = 2.548, *p* = 0.016).

Considering self-esteem, linear regression analysis showed that reduced self-esteem was associated with more severe anxiety (*B* = −0.350, *t* = −2.785, *p =* 0.009) and greater visibility of the symptoms (*B* = −2.638, *t* = −3.004, *p =* 0.006). 

Finally, poorer psychosocial QoL was associated with more severe anxiety (*B*= − 0.920, *t* = −2.286, *p* = 0.030) and more victimization behaviors (*B* = −1.194, *t* = −2.412, *p* = 0.023).

### 3.2. Mediation Analysis

A mediation model was designed to test the possible mediation effect of victimization behaviors on the relationships between psychological symptoms and psychosocial QoL. 

More severe anxiety symptoms were related to more victimization behaviors (*B* = 0.191; *p* = 0.041) but not to cybervictimization behaviors (*B* = 0.056; *p* = 0.115), while no significant relationship emerged between depressive symptoms and victimization (*B* = −0.044; *p* = 0.748) and cybervictimization behaviors (*B* = 0.000; *p* = 0.996). Subsequently, more victimization behaviors (*B* = −1.390; *p* = 0.005) and more depressive symptoms (*B* = −0.873; *p* = 0.024) were related to reduced psychosocial QoL.

**Figure 1:**
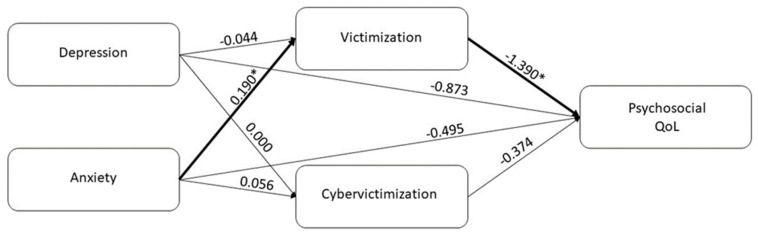
Scheme of the mediation effect of victimization behaviors in the relationship between anxiety and psychosocial quality of life (* *p* < 0.05).

The 95% bias-corrected CI based on 5000 bootstrap samples revealed that the indirect effect of anxiety symptoms on psychological QoL through victimization behaviors was significant (Estimate effect: −0.265; 95% CI: −0.834–−0.016). The absence of a significant direct effect (Estimate effect: −0.495; 95% CI: −1.035–0.044) and the significance of the total effect (Estimate effect: −0.782; 95% CI: −1.328–−0.235) of anxiety symptoms on psychosocial QoL indicate a mediation of victimization behaviors (Figure 1).

## 4. Discussion

In the present study, we explored the occurrence and severity of bullying/victimization behaviors and psychological symptoms in a sample of children and adolescents with NF1. We also examined possible sex differences and the relationship between clinical aspects of NF1 disease and the psychosocial wellness of the patients with NF1.

We found that victimization behaviors were very frequent in our sample of NF1 patients; in fact, about 50% of them experienced victimization at least once in the last six months, while 23.7% experienced victimization several times in a month. These results are in line with the previous study by Holland and colleagues (2019) who reported an almost comparable prevalence rate (25.9%) of victimization behaviors higher than in the general population [33,48]. Cybervictimization behaviors were also frequent in our sample (31.6%) and this prevalence is higher compared to healthy peers [49]. Analyzing bullying instead, we found that patients with NF1 showed less traditional and cyberbullying behaviors (7.9% and 15.8%, respectively) than same school-aged peers ranging from 12.8% [48] to 23.0%, respectively [50].

As for the psychological profile of our NF1 patients, we found the presence of depressive and anxiety symptoms, reduced self-esteem, and worse QoL. Our findings of more severe anxiety and depression and poorer self-esteem and QoL are in line with previous studies revealing differences in psychological symptoms between NF1 patients and healthy subjects [11,13,16,51,52,53]. In particular, prevalence rates of anxiety and depression (18.4 and 5.3%, respectively) in our NF1 cohort were higher compared to same-school-aged peers (ranging from 3 to 6.21% for anxiety and from 1.66 to 2.1% for depression) [54,55].

Taken together, these findings may suggest that children and adolescents with NF1 experience more victimization behavior rather than taking on the role of bullies due to their psychological profile, characterized by higher levels of anxiety and low self-esteem [16,56], which is more compatible with the role of the victim instead of the bully. Moreover, this evidence further supports the presence of psychopathological manifestations in NF1; however, future pathophysiological studies should clarify whether these could be considered intrinsic features of NF1 rather than induced by multisystemic disease or secondary outcomes of the diagnosis.

Subsequently, we investigated the possible existence of gender differences in bullying and victimization experiences revealing differences only in victimization behaviors. In contrast to other studies exploring gender differences in bullying [35,57,58], we found that females complained of more episodes of victimization behavior than males. These results could be affected by the use of the face-to-face interview rather than anonymous self-reported measures, thus leading male participants to be less willing to report their experiences of victimization [59]. Indeed, gender differences in bullying and victimization behaviors often reflect gender socialization, normative expectations as well as the exercise of social power with males experiencing more direct aggressive behaviors (e.g., physical aggressions) compared to females who experience indirect forms of aggression (e.g., exclusion from activities, rumor spreading) [35,57,58].

In addition, we found that females reported more severe anxiety and depression, reduced self-esteem, and poorer psychosocial QoL than males. These results are in line with previous studies in NF1 [14] and the general population [54,60,61] confirming that females are more at risk of developing psychological symptoms in everyday life and stressful situations due to biological vulnerabilities [62,63,64].

Furthermore, we explored the impact of demographic and clinical variables but also of bullying and victimization behaviors on the development of psychological symptoms, QoL, and self-esteem. We found that higher levels of anxiety were associated with female sex, more cybervictimization behaviors, worse depressive symptoms, and lower self-esteem; more severe depression scores were related to higher anxiety; reduced self-esteem was linked to more severe anxiety and more visibility of the NF1 symptoms; finally, poorer psychosocial QoL was linked to more severe anxiety and more victimization behaviors.

Our findings showed that experiencing victimization behaviors, in both face-to-face and cyber modalities, represents a risk factor for more severe anxiety and reduced psychosocial QoL. Considering that NF1 is often linked to anxiety [7,65,66], the occurrence of cybervictimization behaviors seems to exacerbate these symptoms [32,67,68] and escalate the risk of later symptoms as anxiety disorders [69,70]. Indeed, victimization behaviors are frequently reported by individuals who need special health care [71] leading to worse psychological manifestation and reduced QoL.

More specifically, our results indicated that the presence of victimization behaviors reduced the psychosocial QoL of NF1 patients in line with previous studies [23,72,73] and further support that being bullied during childhood and adolescence leads to long-term psychological consequences over the course of life [74,75].

We found that patients’ self-esteem was impacted by the visibility of the NF1 phenotype. In our sample, seven participants reported no visible manifestation of the NF1 disease, and thus we adopted a modified version of Ablon’s scale [39], whereas most of the participants reported aesthetic features that could represent a risk factor for the occurrence of psychological symptoms. Typical NF1 features such as café-au-lait spots, freckles of the skin folds, Lisch nodules in the iris, bone dysplasia, external neurofibromas, and scoliosis [76] are clearly visible causing psychological distress [77,78] and negatively affecting the patients’ perceived body image and self-esteem [79]. In particular, childhood and adolescence are crucial periods for identity formation, and self-esteem is heavily influenced by the perceived body image since it plays a pivotal role in individuals’ self-concept [80].

Moreover, because of these manifestations, people with NF1 might be stigmatized and experience severe psychological symptoms that could severely affect the establishment and maintenance of interpersonal relationships [19,39,81,82]. Taken together, our findings indicate the presence of a maladaptive loop in NF1 patients characterized by psychological symptoms, unfavorable self-perception, low self-esteem, and psychosocial difficulties that might be worsened by experiencing victimization behaviors as indicated by mediation analysis.

The evidence from the literature indicates that NF1 is characterized not only by neurological problems, physical–skeletal defects, visual problems, and hypertension but also by difficulties related to psychosocial well-being with important impacts on patients’ mental health and QoL. Multidisciplinary approaches comprising surgical treatments to reduce the aesthetic impact of NF1-related alterations together with psychotherapy interventions might promote psychological well-being in NF1 patients, especially in crucial stages of the development such as childhood and adolescence [83,84]. Indeed, surgery is often offered as a treatment for severe scoliosis, tibial dysplasia, reduction or removal of plexiform neurofibromas, and excision of cutaneous neurofibromas.

Nevertheless, some limitations of the present study should be addressed. First, the limited number of participants does not allow the generalization of the results. However, it should be considered that NF1 is a rare disease and that we decided to focus on children and adolescent patients further limiting the recruitment. The second limitation might be the absence of a control group since the aim of the present study was to explore the presence and severity of bullying/victimization behaviors in NF1 children and adolescents that represent an at-risk population for psychological consequences. Another limitation might be the adoption of the statements of children and adolescents to investigate bully-victim and cyberbullying/cybervictimization behaviors. The presence of other informants such as parents and teachers could have provided more reliable information. Further studies could overcome this limit by also exploring differences between NF1 patients and their caregivers’ reports.

## 5. Conclusions

We estimated bullying and victimization experiences and evaluated behavioral and psychiatric variables in a cohort of children and adolescents with NF1. Our results confirm the impact of experiencing victimization behaviors on the psychosocial wellness of school-aged patients with NF1, an effect characterized by reduced self-esteem, internalizing disturbances, and difficulties in social interactions.

Therefore, we suggest using a multidisciplinary approach in the diagnosis and treatment of patients with NF1, through the involvement of experienced mental-health clinicians such as psychiatrists and psychologists. Moreover, we highlight the need to develop and implement timely interventions to promote equity and inclusion across social contexts for school-aged individuals with NF1 to avoid negative psychological consequences.

## Figures and Tables

**Table 1:** Comparisons of demographic, clinical, and behavioral variables between males and females.

	Males (*n* = 21)	Females (*n* = 17)	U Mann–Whitney/X²	*p*-Value
Age, years mean (SD)	13.80 (3.00)	13.94 (3.02)	172.00	0.84
OV, mean (SD)	9.61 (9.73)	12.29 (15.74)	173.00	0.86
DHV, mean (SD)	0.04 (0.21)	1.4 (2.98)	132.50	0.06 *
SU-T, mean (%)	7 (35)	5 (29.4)	0.13	0.71
PH-T, *n* (%)	0	3 (17.6)	4.02	0.04 *
NP, *n* (%)	8 (38.1)	4 (23.5)	0.92	0.33
Severity, mean (SD)	1.71 (0.64)	1.82 (0.72)	165.00	0.66
Visibility, mean (SD)	1.42 (0.87)	1.70 (.091)	144.00	0.25
Familiarity, *n* (%)	8 (38.1)	8 (47.1)	0.31	0.57
School support, *n* (%)	5 (23.8)	2 (11.8)	0.90	0.34
VICT, *n* (%)	2 (9.5)	7 (41.2)	5.20	0.02 *
BUL, *n* (%)	1 (4.8)	2 (11.8)	0.63	0.42
CV, *n* (%)	5 (23.8)	7 (41.2)	1.31	0.25
CB, *n* (%)	4 (19)	2 (11.8)	0.73	0.54
CDI, mean (SD)	5.38 (4.09)	10.24 (6.67)	94.50	0.01 *
CDI, *n* (%)	0	2 (11.7)	2.60	0.10
RCMAS, mean (SD)	6.14 (4.17)	18.35 (8.50)	41.00	<0.01 *
RCMAS, *n* (%)	0	7 (41.1)	10.60	<0.01 *
RSES, mean (SD)	23.52 (14.17)	16.41 (4.54)	26.00	0.00 *
RSES, *n* (%)	0	5 (29.4)	7.11	<0.01 *
PedsQoL-PS, mean (SD)	79.31 (12.64)	68.03 (15.37)	101.00	0.02 *

SD = Standard Deviation; *n* = Number of participants; OV = Outpatient Visits; DHV = Day Hospital Visits; SU-T = Surgical Therapy; PH-T = Pharmacological Therapy; NP = Neuropsychiatric comorbidity; VICT = Victimization; BUL = Bullying; CV = Cybervictimization; CB = Cyberbullying; CDI = Children’s Depression Inventory-2; RCMAS = Revised Children’s Manifest Anxiety Scale-2; RSES = Rosenberg Self-Esteem Scale; PedsQoL-PS = Psychosocial Pediatric Quality of Life; * = significative difference (alpha level < 0.05) between two groups.

## Data Availability

The authors declare that all data used in the conduct of the analyses are available within the article and tables and figures. To protect the privacy and confidentiality of patients in this study, clinical data are not publicly available in a repository or in the Supplementary Materials of the article, but they can be made available upon reasonable request to the corresponding author. Those requests will be reviewed by a study steering committee to verify whether the request is subject to any intellectual property or confidentiality obligations. All data shared will be de-identified.

## Supplementary Materials

The following supporting information can be downloaded at: https://www.mdpi.com/article/10.3390/children10020330/s1, Supplementary Material Table S1: Modified Ablon scale.
