# Supplementary material for: Neuropsychiatric Manifestations, Reduced Self-Esteem and Poor Quality of Life in Children and Adolescents with Neurofibromatosis Type 1 (NF1): The Impact of Symptom Visibility and Bullying Behavior
Source: Children (Basel). 2023 Feb 9;10(2):330. doi: 10.3390/children10020330 (PMC9955783; doi:10.3390/children10020330)
Supplement: Supplementary file 1 [file children-10-00330-s001.zip › children-2196759-supplementary.pdf]

Supplementary Materials Table S1. Modified Ablon scale to clinically assess the severity and visibility of NF1 disease manifestations.

**Severity:** the combination of clinical and cosmetic implications which may potentially affect lifestyle, mobility and/or threaten life.	
**1. Mild**	Neurofibromas or mild learning and/or speech disorders which do not threaten physical or social life; mild scoliosis.
**2. Moderate**	Numerous external and internal neurofibromas, moderate scoliosis, learning and/or speech disorders that compromise social interaction and lifestyle;
**3. Severe**	Neurofibromas that threaten functioning; serious internal neurofibromas; malignancies; optic glioma; severe scoliosis or other abnormal severe skeletal features.

**Visibility:** concerns the appearance of the person fully dressed and how readily symptoms could be perceived in impersonal interaction; café-au-lait spots and freckling haven't been considered, because of their irrelevant role in impersonal and interpersonal interaction.	
**1. None**	No neurofibromas all over the body; gate and posture unremarkable when casually observed; no scoliosis or other abnormal skeletal features; no dysmorphism.
**2. Mild**	No visible cutaneous and subcutaneous neurofibromas outside normal clothing areas. However, it should be noted that many people who have no tumors on visible areas, have numerous neurofibromas on the chest, abdomen, pelvis or thigh which would be remarkable in intimate situations and that may severely affect sexual behavior; mild scoliosis or scoliotic attitude or other skeletal features not perceived in impersonal interaction through abnormal gate and posture.
**3. Moderate**	Some neurofibromas on the neck, face, hand, forearm, leg or detectable in impersonal interaction because of modification of normal physical appearance; moderate scoliosis or other abnormal skeletal features visible in impersonal interaction and intimate situations; in this class are included dysmorphisms; short stature (<3°).
**4. Severe**	Numerous neurofibromas on the n face; severe scoliosis or skeletal features with a noticeable limp; optic glioma that has affected sight and eye socket.
